# Diversified Biomineralization Roles of *Pteria penguin* Pearl Shell Lectins as Matrix Proteins

**DOI:** 10.3390/ijms22031081

**Published:** 2021-01-22

**Authors:** Tomohisa Ogawa, Rie Sato, Takako Naganuma, Kayeu Liu, Saho Sato, Shizuka Sakaue, Makoto Osada, Kyosuke Yoshimi, Koji Muramoto

**Affiliations:** 1 Graduate School of Agricultural Science, Tohoku University, Sendai 980-8572, Japan; makoto.osada.a8@tohoku.ac.jp; 2 Graduate School of Life Sciences, Tohoku University, Sendai 980-8577, Japan; riesato310@gmail.com (R.S.); naga@mishima.ac.jp (T.N.); knight.lky@gmail.com (K.L.); saho_land@me.com (S.S.); sakaue.2013@gmail.com (S.S.); kojimuramoto@yahoo.co.jp (K.M.); 3 Center for Interdisciplinary Research, Tohoku University, Sendai 980-8578, Japan; yoshimi@material.tohoku.ac.jp; 4 Department of Materials Science, Graduate School of Engineering, Tohoku University, Sendai 980-8579, Japan

**Keywords:** biomineralization, carbohydrate, chitin, lectin, pearl shell

## Abstract

Previously, we isolated jacalin-related lectins termed PPL2, PPL3 (PPL3A, 3B and 3C) and PPL4 from the mantle secretory fluid of *Pteria penguin* (Mabe) pearl shell. They showed the sequence homology with the plant lectin family, jacalin-related β-prism fold lectins (JRLs). While PPL3s and PPL4 shared only 35%–50% homology to PPL2A, respectively, they exhibited unique carbohydrate binding properties based on the multiple glycan-binding profiling data sets from frontal affinity chromatography analysis. In this paper, we investigated biomineralization properties of these lectins and compared their biomineral functions. It was found that these lectins showed different effects on CaCO_3_ crystalization, respectively, although PPL3 and PPL2A showed similar carbohydrate binding specificities. PPL3 suppressed the crystal growth of CaCO_3_ calcite, while PPL2A increased the number of contact polycrystalline calcite composed of more than one crystal with various orientations. Furthermore, PPL4 alone showed no effect on CaCO_3_ crystalization; however, PPL4 regulated the size of crystals collaborated with *N*-acetyl-D-glucosamine and chitin oligomer, which are specific in recognizing carbohydrates for PPL4. These observations highlight the unique functions and molecular evolution of this lectin family involved in the mollusk shell formation.

## 1. Introduction

Biomineralization is the biological process of crystallization of inorganic materials under the strict biocontrol by living organisms. They include bones, teeth, eggshells, mollusc shells, corals, and coccolithophore, which are organic-inorganic nano-composite composed of organic matrices such as proteins and polysaccharides. Mother of pearl is one sophisticated organic-inorganic hybrid material composed of CaCO_3_ aragonite crystals arranged in multi-layered mineral lamellae. Pearl oysters can produce two types of calcium carbonate crystals concurrently and specially: calcite on the prismatic structured layer and aragonite on the nacreous layer. The winged pearl oyster (*Pteria penguin*) occurs naturally in the tropical Southeast or West Pacific Ocean coastlines, and is one of the important species for pearl aquaculture to provide the large half-round pearls. While some studies have investigated the biomineralization-related genes in *P. penguin* at the transcriptomic level [1,2], the molecular mechanisms involved in nacre formation and regulation are almost unknown. Previously, we isolated and characterized jacalin-related β-prism fold lectins termed PPL2A, PPL2B, PPL3s and PPL4 from the secretory fluid of *P. penguin* mantle [3,4]. These lectins shared 35%–50% homology each other and showed only 20%–27% homology to jacalin. Lectins are nonenzymatic proteins that bind to carbohydrates specifically and regulate the biological roles of cells, carbohydrates and proteins. Both PPL2A and PPL2B regulated the morphological populations of calcite crystals in vitro [3], suggesting that lectins from the secreted fluid of *P. penguin* are involved in shell formation. Recently, their carbohydrate-binding properties were analyzed by frontal affinity chromatography analysis, resulting in multiple glycan binding profiling data sets for PPL2A, PPL3 and PPL4. These data revealed that the carbohydrate-binding specificity of PPL3s was similar to that of PPL2A, except only for Man3Fuc1Xyl1GlcNAc2 (M3FX) *N*-linked oligosaccharide, while PPL4 showed the different carbohydrate-binding properties compared to PPL2A and PPL3s. PPL2A and PPL3s can recognize both agalactosylated and galactosylated-type glycans, while PPL4 binds to high-mannose and hybrid-type *N*-linked glycans, but not agalactosylated and galactosylated-type glycans [4]. PPL4 can also recognize chitin oligomers [4], of which polymers are well known as components of the shell matrix. Furthermore, we recently determined the 3D structures of PPL3s by X-ray crystallography, including the free lectin forms and lectins complexed with trehalose and isomaltose, respectively [5]. Docking simulations of PPL3 to various calcite crystal faces based on its 3D structure also revealed the charged amino acid residues located on the edge of a β-sheet and the carbohydrate-binding site can contribute to the interaction with calcite [5].

In this study, to clarify the biological functions and relevance of *P. penguin* multiple lectins and their ligand carbohydrates to biomineralization, we investigate the effects of two jacalin-related lectins, PPL3 and PPL4, on CaCO_3_ crystalization and shell formation, and compared with those of PPL2A and PPL2B. These lectins showed the different uneque biomineralization activities of each other with/without carbohydrates.

## 2. Results

### 2.1. Knockdown Analysis of PPL3 and PPL4 in the Biomeneralization Processes

**Figure 1:**
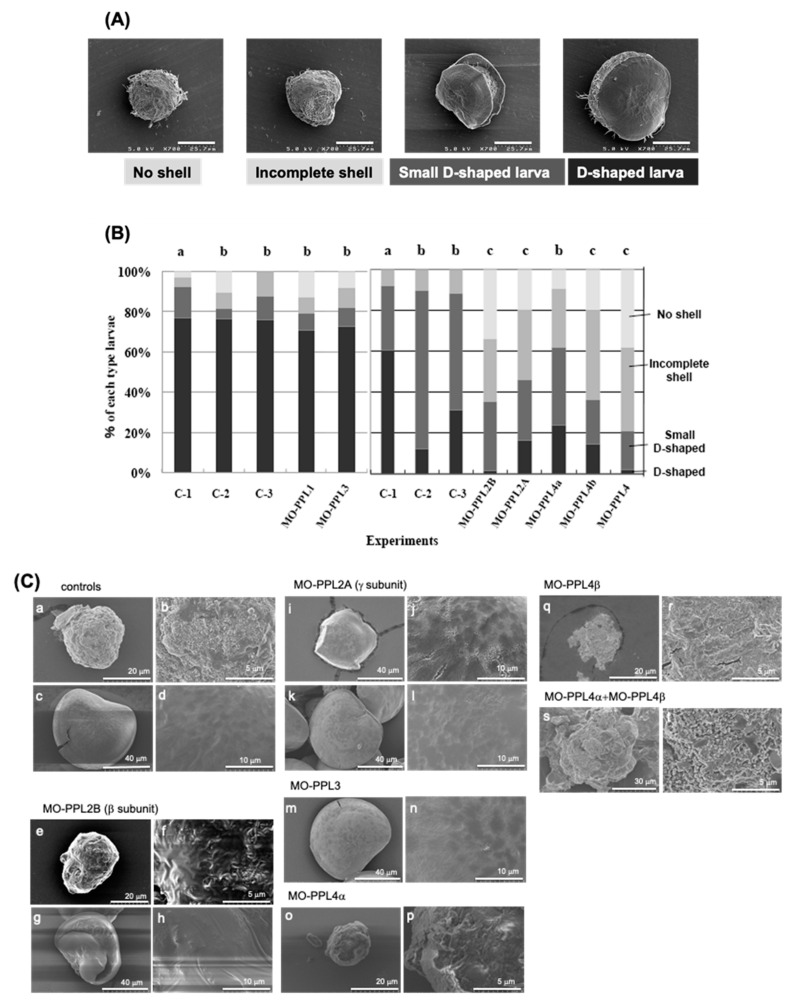
Morphological effects of knockdown of PPL2A, PPL2B, PPL3 and PPL4 on D-shaped shell formation at larval stage. (**A**) Typical SEM images of D-shaped larvae with or without morpholino oligos at a ×700 magnification. Larva phenotypes could be divided into four groups, no shell, incomplete shell, small D-shaped larva, and D-shaped larva. Scale bars indicate 25.7 μm. (**B**) Frequency of the phenotypes obtained by knockdown analysis of PPL3 and PPL4 in addition to PPL1, PPL2A, and PPL2B. MO-PPL1: treated with morpholino oligo for PPL1, MO-PPL2B: treated with morpholino oligo for PPL2B (β subunit), PPL2A: treated with morpholino oligo for PPL2A (γ subunit), MO-PPL3: treated with morpholino oligo for PPL3, MO-PPL4α: treated with morpholino oligo for PPL4α, MO-PPL4β: treated with morpholino oligo for PPL4β, MO-PPL4: treated with both morpholino oligos (MO-PPL4α and MO-PPL4β) for PPL4α and PPL4β. Two independent experimental groups were conducted with three controls, C-1: no regent, C-2: treated with Endo-porter, and C-3: treated with Endo-porter and control oligos (10 nM), respectively. Each experiment was conducted in duplicate and analyzed using chi-square tests (df = 2). **a**: criteria samples, **b**: *p* > 0.005, **c**: *p* < 0.001. (**C**) Typical SEM images of larvae knockdown by MO-PPL2B (**e**,**f** for incomplete shell; **g**,**h** for small D-shell), MO-PPL2A γ subunit (**i**,**j** for small D-shell; **k**,**l** for D-shell), MO-PPL3 (**m**,**n** for D-shell), MO-PPL4α (**o**,**p** for no shell), MO-PPL4β (**q**,**r** for no shell), MO-PPL4α + MO-PPL4β (**s**,**t** for no shell), and controls (**a**,**b** for normal blastulae; **c**,**d** for normal D-shell) with ×1.20 k (**c**,**g**,**i**,**k**,**m**), ×1.50 k (**s**), ×2.0 k (**e**), ×2.50 k (**a**,**o**,**q**), ×5.00 k (**d**,**h**,**j**,**l**,**n**), and ×10.0 k (**b**,**f**,**p**,**t**) magnifications.

To analyze the direct involvement of the *P. penguin* lectins, PPL3s and PPL4, within the biomineralization process, the knockdown experiments using morpholino oligos (MOs), which can be suppressed by the expression at the translational level, were conducted and compared with PPL2A and PPL2B, of which knockdown experiments and in vitro crystallization have been previously reported [3]. After introducing the MOs of each gene, the rate of D-shell formation for each experiment was estimated by counting four morphological types of larva at 48 h post fertilization (hpf), that is D-shaped shell larva (D-shell), small D-shaped shell larva, incomplete shell and no shell, which were determined from SEM micrographs (Figure 1A). Two independent knockdown experiments using different *P. penguin* larvae sets prepared from different individual were conducted by using each MO with respective controls (C-1, C-2 and C-3), respectively (Figure 1B left and right panels). As the sizes of D-shells in the controls of the second experiment (right panel of Figure 1B) were slightly smaller than those in the first experimental controls (Figure 1B); knockdown experiments were assessed by counting the number of combined D-shell and small D-shell larvae. The percentage of complete D-shaped larva, including small size, was similar among three control experiments (84%–92%), although the second experiment including PPL2 and PPL4 (right panel of Figure 1B) showed the larger numbers of small size but distinct D-shaped larva than the other (left panel). These results indicated that there was no effect of Endo-porter and/or control MOs on the morphology during *P. penguin* larva development, although some interexperimental differences were detected on the size of D-shaped larva (Figure 1B).

Knockdown analysis for D-shell structure formation at the larval stage showed that PPL4 α and β subunits have significant effects on D-shell formation the same as PPL2A (PPL2γ subunits) and PPL2B (PPL2β subunit)—that is, 78.7% of larva treated with MO-PPL4 (PPL4α and PPL4β) had no shell (38.3%) and incomplete shell (40.4%); only 21.2% had small or normal D-shaped shell formation (Figure 1B). D-shell formation of larva was more affected by MO-PPL4β-treated (63.6% of no or incomplete shell) than MO-PPL4α (38.1% of no or incomplete shell). However, the knockdown of PPL3 showed no effect on D-shell formation, which is the same as PPL1 (Figure 1B). Furthermore, to elucidate the direct effects of PPL3 and PPL4 in addition to PPL2A and PPL2B on D-shell formation in more detail, microimages of larva treated with each MO were analyzed by SEM and compared morphologies with each other (Figure 1C). Knockdown of PPL2B showed the severe damage and defect for shell formation such as incomplete shell (Figure 1C(e,f)) and largely distorted shell (Figure 1C(g,h)). Interestingly, knockdown of the PPL2A γ subunit brought on cracks along the lines with the boundary face of crystal grain in both small D-shell (Figure 1C(i,j)) and D-shell (Figure 1C(k,l)). Knockdown of PPL3 showed no effect on D-shell apparently (Figure 1C(m)); however, it increased surface fine roughness (Figure 1C(n)) compared to controls (Figure 1C(d)). Knockdown of PPL4α and β subunits caused defects on D-shell formation at the early level, although some matrix layer and small crystals were observed on the surface of larvae (Figure 1C(o–t)).

### 2.2. Molecular Properties of PPL3 and PPL4 on In Vitro Crystallization

**Figure 2:**
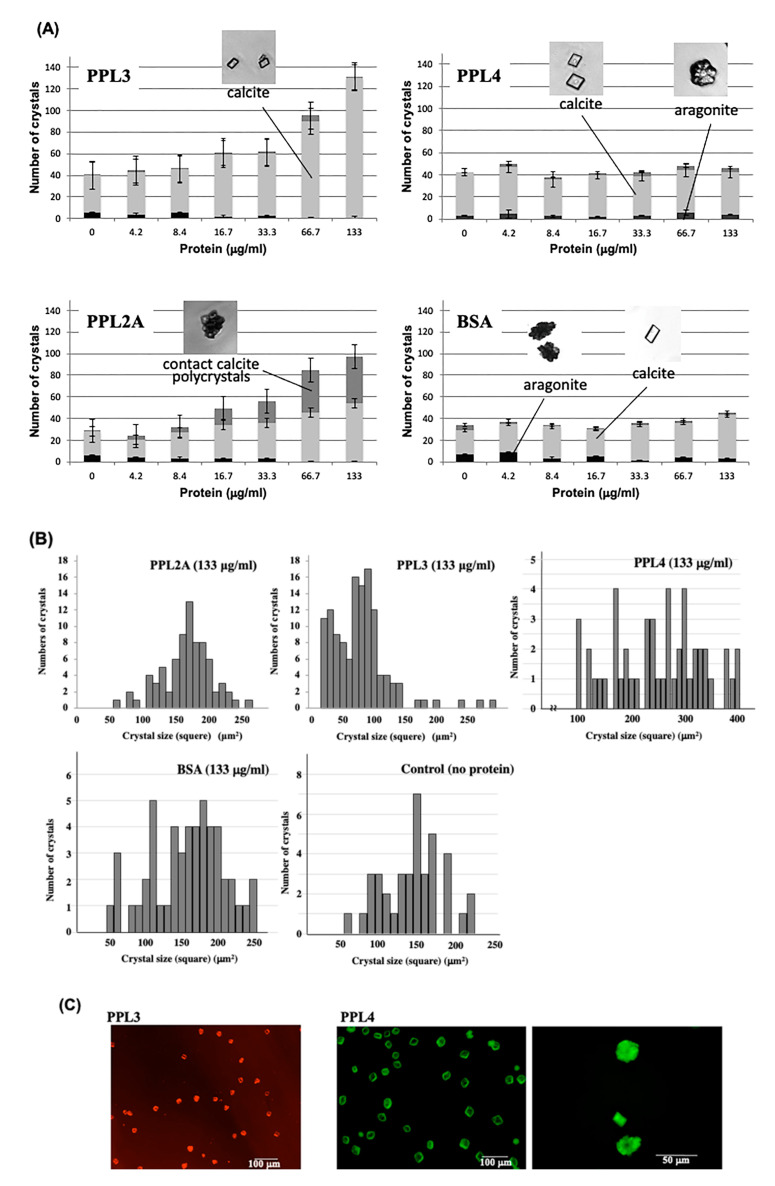
Effects of PPL2A, PPL3 and PPL4 on the in vitro CaCO_3_ crystallization. (**A**) Effects of PPL3 and PPL4 on the number and morphology of CaCO_3_ crystals, i.e., calcite, calcite polycrystal, and aragonite. Bovine serum albumin (BSA) and PPL2A [3] were used as negative and positive controls, respectively; (**B**) Effects of PPL2A and PPL3 on the crystal size. Distribution of crystal size (square) was estimated by using MetaMorph software (Molecular Devices, LLC, San Jose, CA, USA); (**C**) Fluorescence microscopy imaging of Alexia568-labeled PPL3 (red) and Alexia488-labeled PPL4 (green) on CaCO_3_ crystallization.

In vitro crystallization experiments using PPL3 and PPL4 were also conducted and the crystal numbers and forms were compared to those of PPL2A. Optical microscope images of in vitro crystallization showed that the number of calcites increased depending on the concentration of PPL3, while PPL4 showed no effect on the morphology and the number of crystals (Figure 2A). However, PPL2A was repeatedly shown to increase the rate of polycrystalline CaCO_3_ in a dose-dependent manner, which is the same as shown in previous reports (Figure 2A) [3]. Furthermore, the crystal sizes in the presence of PPL3 were smaller than those of PPL2A and BSA (used as a control) at a concentration of 133 μg/mL, indicating that PPL3 regulated the crystal growth of calcite by suppressing (Figure 2B). However, the crystal sizes in the presence of PPL4 were slightly larger, as compared to controls, BSA and no proteins (Figure 2B).

In order to further elucidate how PPL3 and PPL4 affect the growth of crystals, their distributions in crystal formation were investigated using the fluorescence-labeled PPL3 and PPL4, respectively (Figure 2C). During crystallization with AF568-labeled PPL3 (red), PPL3 was located at the surface of crystals (Figure 2C), suggesting that PPL3 inhibited the calcium carbonate crystallization by binding to the crystal growth surface. However, AF488-labeled PPL4 (green) was located at the interior aragonite in addition to the surface of calcite crystals (Figure 2C), although PPL4 alone showed no effect on the number of crystals (Figure 2A).

### 2.3. Effect of Carbohydrates on the Biomeneralization Processes of PPL3 and PPL4

**Figure 3:**
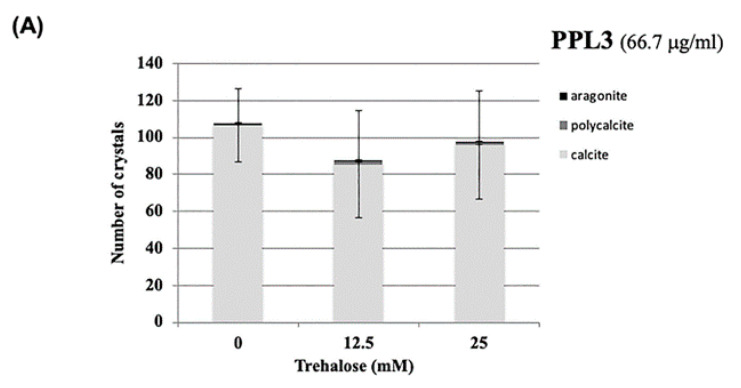

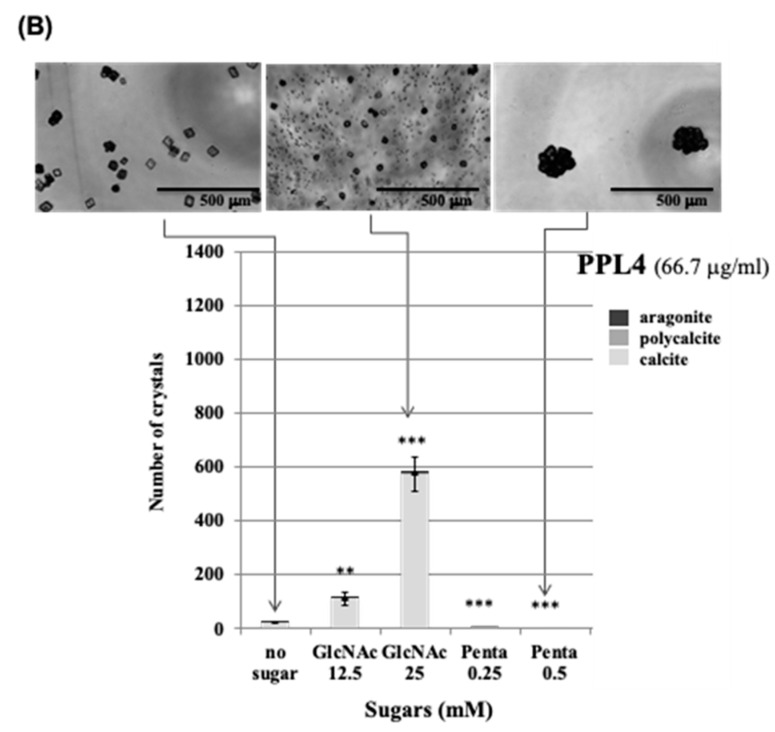
Effects of carbohydrates on the CaCO_3_ crystallization in the presence of PPL3 (**A**) and PPL4 (**B**). Trehalose (treha), *N*-acetyl-d-glucosamin (Glc*N*Ac), and penta-*N*-acetyl-chitopentaose (Penta) were used at indicated concentrations (mM), respectively, with the same concentration (66.7 mg/mL) as PPL3 and PPL4. Micrographic images of crystals with or without carbohydrates at a ×4 magnification. The data were evaluated by using *t*-test. ***: *p* < 0.001, **: *p* < 0.005.

Previously, we found that trehalose, which is abundant in the secretary fluid of *P. penguin* and specifically recognizes carbohydrates for PPL2A, can regulate the biomineralization via PPL2A [3]. Furthermore, a comprehensive study of carbohydrate binding specificities of PPL2A, PPL3 and PPL4 revealed that PPL4 can also recognize chitin oligomers, of which polymers are well known as components of the shell matrix [4]. Based on these previous studies, to further elucidate the carbohydrate functions on the CaCO_3_ crystallization, the effects of trehalose, *N*-acetyl-D-glucosamine (Glc*N*Ac), and chitin oligomer that are specific sugars for PPL3 or PPL4, respectively, were assessed in vitro (Figure 3). PPL3 had no additive effect by trehalose, which is different from PPL2A (Figure 3A). Most interestingly, PPL4 was greatly affected by Glc*N*Ac and penta-*N*-acetyl chitopentaose (one of chitin oligomers), although PPL4 alone has no or a small effect on CaCO_3_ crystalization (Figure 3B). Thus, the specific carbohydrates such as Glc*N*Ac increased the number of calcite crystals and decreased the crystal sizes in the presence of PPL4. However, penta-*N*-acetylchitopentaose oligosaccharide increased the size of polycrystals (186 μm × 157 μm) with PPL4, although the crystal number was markedly decreased (Figure 3B). These results suggest that PPL4 are regulated by sugars, resulting in considerably different biomineralization functions, which can either suppress or enhance the crystal growth.

## 3. Discussion

Two jacalin-related lectins, PPL3 (3A, 3B, 3C) and PPL4, isolated from the secreted fluid of mantle of *Pteria penguin* pearl shells, were characterized as matrix proteins of pearl shell in addition to PPL2A and PPL2B [3,4]. Knockdown of PPL4 expression caused severe defects in D-shaped shell formation during larva development in vivo, which was the same as PPL2A and PPL2B [3] (Figure 1B). In particular, knockdown of both PPL4 α and β subunits showed the severe defects and additive effect on D-shell formation at the early level (Figure 1C). In in vitro assay for crystallization, although PPL4 alone showed no or small effect on the number of crystals without carbohydrate (Figure 2A), PPL4 increased the crystal sizes, including aragonite-like crystals (Figure 2B,C). These properties and effects of PPL4 on the in vitro CaCO_3_ crystallization were similar to that of PPL2B, as previously reported [3]. Furthermore, PPL4 showed the unique biomineralization properties in combination with specific carbohydrates such as chitin oligomer and Glc*N*Ac—that is, PPL4 increased the size of polycrystals with chitin oligomer (Figure 3B). Chitin is well known as a key component of the extracellular matrix of mollusk shells produced by chitin synthases [6,7,8], and highly ordered at the molecular level responsible for the interlamellar structure that was proposed as a matrix model of chitin-silk fibroin gel proteins–acidic macromolecules [9,10], including the nacreous layer [11,12,13,14] and regulates calcite nucleation [15]. Furthermore, it has been reported that the polysaccharides that contain high levels of carboxylate and sulfate also regulate (promote) the growth and morphology of calcite crystals [16]. Chitin synthases have also been found in transcriptome data of *P. penguin* reported by Li et al. [2]. Additionally, numerous examples of chitin-binding proteins that show important roles on biomineralization involved in nacreous layer formation such as Pif-like [17,18], pearlin/N16 family [19], Hichin [20] and PS19 [21], etc., have been reported. Taken together with PPL4 activity with chitin oligomer, PPL4 may participate in the biomineralization process of *P. penguin* nacre and the D-shell formation from an early stage as chitin-binding proteins and calcium carbonate crystallization regulators.

However, we have previously reported that PPL2A and PPL2B contribute directly to biomineralization [3]. Specifically, PPL2A increased the number of contact polycrystalline calcite by binding to the boundary regions of crystals or the emergence region of the polycrystalline on the nacreous layer, while PPL2B induced the flower petal-like shape aragonite crystals [3]. In this study, interestingly, we found that the knockdown of PPL2A γ subunit brought on the cracks along the lines with a boundary face of crystal grain in both small D-shells and D-shells (Figure 1C(i–l)). These results agree well with previous observations, including PPL2A distribution at the boundary region of crystals, and confirm that PPL2A functions as a glue for crystal grain.

**Figure 4:**
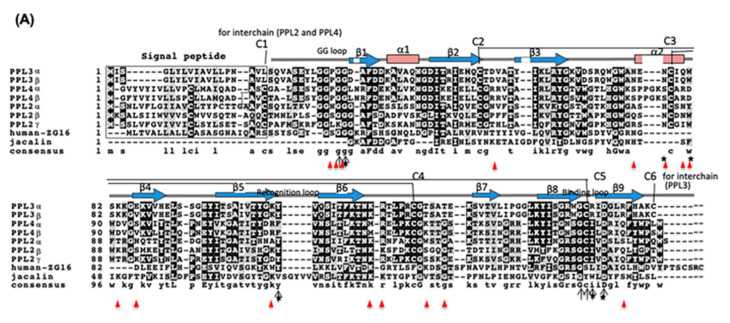

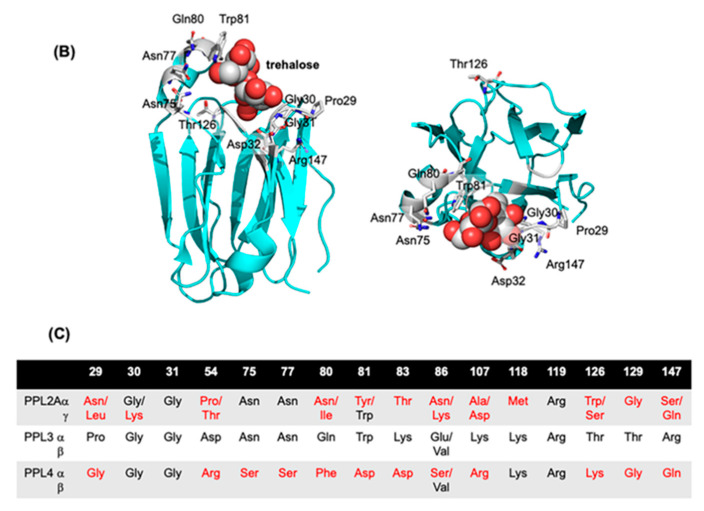
Aligned amino acid sequences of PPL subunits with jacalin-related lectins (**A**) and schematic structure of PPL3 and a comparison of the amino acid residues found at the interfaces of PPL3 to calcite crystal faces among PPL2A, PPL3 and PPL4 (**B**,**C**). The sequences were aligned using Clustal W program and represented by using BOXSHADE 3.21. The residues identical to the column-consensus were presented by inverse character (black background), while the residues, which are not identical but at least similar to the column-consensus, were presented by gray background. C1 to C5 indicates the half cysteine residues with inter and intra-disulfide bonds. Secondary structural elements (β strands) are shown as arrows (β1–β12). The GG loop, recognition loop, and binding loop indicated by arrows were parts of the carbohydrate recognition domain. Red-colored arrowheads indicate the residues found at the interfaces to calcite faces analyzed by the docking study [5].

Conversely, knockdown of PPL3 had no apparent effect on larva development (Figure 1B); however, it increased the surface fine roughness compared to controls (Figure 1C). In vitro crystallization experiments indicated the significant effects on the number of calcite crystals and crystal sizes in the presence of PPL3—that is, the smaller crystals were increased in a concentration dependent manner, indicating that it might suppress and control the growth of calcite crystals by binding to the crystal growth surface (Figure 2). The docking simulations of PPL3B to calcite crystals clearly suggested that its interaction on the calcite crystal surface is comprised of negatively charged residues, Asp32 and Glu86, interacting with calcium ions, and the positively charged residues, Lys83, Lys107, Lys118, Arg119, and Arg147 binding to carbonates on the crystal faces (Figure 4) [5]. Suppression factors of calcite formation seem to be crucial to aragonite formation on nacre such as perlwapin and perlinhibin isolated from abalone nacre as inhibitors of calcite growth, respectively [22,23].

Thus, the multiple JRL isoforms of *P. penguin* pearl shells, PPL2A, PPL2B, PPL3 and PPL4, showed, respectively, the unique and different biomineralization activities with/without carbohydrate and roles directly involved in nacreous pearl shell formation. These carbohydrate-depending biomineralization properties may be due to a possible involvement of residues found in PPL3 at the interfaces to calcite faces, which were closed to the carbohydrate binding site.

In recent decades, the genome, transcriptome and proteome analyses for several pearl oyster species have been achieved using next generation sequencing technology and/or MS/MS technology to identify the key players in nacre formation and to understand the molecular mechanisms of biomineralization [24,25,26,27,28,29,30,31]. In this study, we characterized lectins termed PPL3 and PPL4, in addition to PPL2A and PPL2B, which contribute to the biomineralization processes of *P. penguin* nacreous shells with different carbohydrate recognition profiles. We also found that carbohydrates such as Glc*N*Ac and chitin oligomers can function as regulatory factors for biomineralization coordination with PPLs. While the common nacre matrix proteins such as Pif-like, schematrin-like, Naclein-like, MSI60, and carbonic anhydrase have been identified in molluscan shells, jacalin-like lectins were specifically only identified in *P. penguin*. These observations highlight the unique functions and molecular evolution of this lectin family and their specific-carbohydrates, which work collaboratively in mollusk shell formation.

## 4. Materials and Methods

### 4.1. Materials

Large-winged pearl-shells (*Pteria penguin*, 6-years-old) were provided by Amami South Sea and Mabe pearl Co. Ltd. (former Amami-branch, Tasaki & Co. Ltd.), Kagoshima, Japan. The mantle and its secretory fluid were collected and stored at −80°C or −30°C until use, respectively. A POROS^®^ HS column was purchased from Applied Biosystems, Thermo Fisher Scientific KK (Tokyo, Japan). Resource S and HiTrap NHS activated columns were purchased from GE Healthcare (Chicago, IL, USA). Trehalose was purchased from Hayashibara (Okayama, Japan). All other reagents were of the purest grade commercially available.

### 4.2. Isolation of Lectins from Secretory Fluid of Mantle

The protein samples of PPL2A, PPL2B, PPL3 and PPL4 were prepared from the secretory fluid of the mantle of *Pteria penguin*, as previously reported [4]. In brief, secretory fluid of the mantle was dialyzed against desalinized water. After centrifuge at 10,000 rpm, the supernatant was fractionated by HPLC using cation exchange chromatography on a POROS HS column equilibrated with 10 mM MES/HEPES buffer (pH 6.5) and eluted with a linear gradient of 0 to 1 M NaCl. The eluted fractions were further purified by cation exchange chromatography on a Resource S column pre-equilibrated with 20 mM MES buffer (pH 7.0) and eluted with a linear gradient of 0 to 2 M NaCl in the same buffer, after being dialyzed against water, respectively. Finally, purified PPLs were checked by SDS-PAGE.

### 4.3. Morpholino Design and Morphological Phenotype Analysis of Morphants of P. penguin D-Shaped Larva

The knockdown experiments using morpholino oligos were conducted by reference to the previous papers for invertebrate embryos. Translational-blocking morpholino oligos (MOs) of *P. penguin* lectins, MO-PPL2A, MO-PPL2B, MO-PPL3, MO-PPL4α, and MO-PPL4β corresponding to the antisense nucleotides from the initiator codons of PPL2A γ subunit, PPL2B, PPL3 and PPL4 α and β subunits were designed to block their translation, respectively, and were synthesized using Gene Tools (Philomathe, OR, USA). MO-PPL1 was designed for PPL1 as control. These sequences are as follows: 5′-ACATAAGCACAGCTATCACCAACAT-3′ for MO-PPL1, 5′-AAATAATAGACAAGGCCGATTTCAT-3′ for MO-PPL2A (γ subunit), 5′-TCAGTCCTCCAAAGGTCTTCGACAT-3′ for MO-PPL2B, 5′-CTACCAGATATAGTCCAGAAATCAT-3′ for MO-PPL3, 5′-ACACATAGACACCCATTCTGCTGGT-3′ for MO-PPL4α, and 5′-ACACATAAGAAACCCATTCCGCTGGT-3′ for MO-PPL4β. Standard control morpholino oligo, 5′-CCTCTTACCTCAGTTACAATTTATA-3′, was from Gene Tools. After fertilization of *P. penguin* eggs, floating blastulae were selected by decantation to synchronize the embryonic development. MO-PPL2A, MO-PPL2B, MO-PPL1 and control oligo were dissolved in nuclease-free water at 0.2 mM, and were transfected into synchronized *P. penguin* larvae at a final concentration of 10 mM for each oligo per larvae (5 × 10^3^ population/mL sterilized artificial sea water) at late trochophore larval stage by Endo-porter (8 mM). After incubation at 20°C overnight, the morphants and control D-shaped larvae (treated with or without Endo-porter only) were fixed by adding 20 mL of paraformaldehyde (final 2%) for SEM examination. SEM images of D-shaped larvae were acquired using Hitachi S-4200 and SU-8000 at an acceleration voltage of 5.0 kV and 3.0 kV. Phenotypes of morphants (ca. 50 to 100) were counted and scored according to the relative percentage of 4 morphological types (D-shaped, small D-shaped, incomplete shell, and no shell larvae). Each experiment was conducted in duplicate and analyzed using chi-square tests.

### 4.4. In Vitro Crystallization

The in vitro crystallization experiments were carried out in 96-well titer plates (Non-binding surface, #3881, Corning Inc., New York, NY, USA) by adding PPL3 or PPL4 to the artificial seawater (0.5 M NaCl and 0.011 M KCl), including 10 mM CaCl_2_ and 8 mM NaHCO_3_. The plate was kept for 12 h, 24 h and 48 h at room temperature, and the morphological effects of proteins and carbohydrates on the crystallization of CaCO_3_ were monitored at each interval using an optical microscope. The morphology, size, and number of crystals formed in the presence of proteins was compared to those of crystals grown in parallel without protein prepared as a negative control. Bovine serum albumin (BSA) was also used as a negative control. The effects of trehalose, *N*-acetyl-D-glucosamine, and *N*-acetyl chitopentaose (one of chitin oligomer), which are specific to PPL3 and PPL4, on the crystallization of CaCO_3_ were also assessed. The number and sizes of crystals were estimated using MetaMorph software (Molecular Devices, LLC, CA, USA). The polycrystalline was counted and measured as one polycrystalline entire form but not multiple subunits. Furthermore, the localization of PPL3 and PPL4 on CaCO_3_ crystals was analyzed by using Alexa Fluor 568 (AF568)-conjugated PPL4 (red) and AF488-conjugated PPL3 (green), respectively. Labeling of PPL3 and PPL4 (500 μg each) was conducted using AF568 carboxylic acid 2,4,5,6-Tetrafluorophenyl (TFP) ester or AF488 TFP ester, according to the manufacturer’s protocols (Life Technologies Co., Carlsbad, CA USA). After samples were purified by gel filtration and concentrated by ultrafiltration, AF568-conjugated PPL3 and AF488-conjugated PPL4 were used for in vitro crystallization experiment. Localization of PPL3 and PPL4 on crystals was imaged via fluorescence microscopy (Olympus IX71 equipped with Hamamatsu Digital camera C10600 ORCA-R2) using MetaMorph NX software. A mercury lamp with U-MNIBA3 filter (Ex 470–495 nm, Em 510–550 nm, dichroic filter 505 nm) and U-MWIG3 filter (Ex 530–550 nm, Em 575 nm, dichroic filter 570 nm) were used for fluorescence imaging.

## 5. Conclusions

In the present study, we found unique functionally distinct properties of divergent lectins termed PPL3 and PPL4, in addition to PPL2A and PPL2B, which contribute to the biomineralization processes of *P. penguin* nacreous shells. We also found that carbohydrates such as Glc*N*Ac and chitin oligomers can function as regulatory factors for biomineralization coordination with PPLs. These observations highlight the unique biomineralization functions and molecular evolution of this lectin family and their specific-carbohydrates, which work collaboratively in mollusk shell formation.

## Data Availability

Data is contained within the article.

## Abbreviations

A	Alexa Fluor
BSA	bovine serum albumin
CRD	carbohydrate recognition domain
Fuc	fucose
Glc*N*Ac	*N*-acetyl-D-glucosamine
HPLC	high performance liquid chromatography
JRL	jacalin related lectin
Man	mannose
MO	morpholino oligo
MS	mass spectrometry
SDS-PAGE	sodium dodecyl sulfate polyacrylamide gel electrophoresis
SEM	scanning electron microscopy
Xyl	xylose
M3FX	Manαl~6(Manαl~3)(Xylβl~2)Manβl~4GlcNAcβ1~4(Fucαl~3)GlcNAc-

## References

[1] Huang X.D., Zhao M., Liu W.G., Guan Y.Y., Shi Y., Wang Q., Wu S.Z., He M.X. (2013). Gigabase-scale transcriptome analysis on four species of pearl oysters. Mar. Biotechnol..

[2] Li H., Liua B., Huang G., Fan S., Zhang B., Su J., Yu D. (2017). Characterization of transcriptome and identification of biomineralization genes in winged pearl oyster (*Pteria penguin*) mantle tissue. Comp. Biochem. Physiol. Part D.

[3] Naganuma T., Hoshino W., Shikanai Y., Sato R., Liu K., Sato S., Muramoto K., Osada M., Yoshimi Y., Ogawa T. (2014). Novel matrix proteins of *Pteria penguin* pearl oyster shell nacre homologous to the Jacalin-related β-prism fold lectins. PLoS ONE.

[4] Ogawa T., Sato R., Naganuma T., Liu K., Lakudzala A.E., Muramoto K., Osada M., Yoshimi K., Hiemori K., Hirabayashi J. (2019). Glycan Binding Profiling of Jacalin-Related Lectins from the *Pteria penguin* Pearl Shell. Int. J. Mol. Sci..

[5] Nakae S., Shionyu M., Ogawa T., Shirai T. (2018). Structures of jacalin-related lectin PPL3 regulating pearl shell Biomineralization. Proteins.

[6] Suzuki M., Sakuda S., Nagasawa H. (2007). Identification of chitin in the prismatic layer of the shell and a chitin synthase gene from the Japanese pearl oyster, *Pinctada fucata*. Biosci. Biotechnol. Biochem..

[7] Weiss I.M., Schönitzer V., Eichner N., Sumper M. (2006). The chitin synthase involved in marine bivalve mollusk shell formation contains a myosin domain. FEBS Lett..

[8] Weiss I.M., Lüke F., Eichner N., Guth C., Clausen-Schaumann H. (2013). On the function of chitin synthase extracellular domains in biomineralization. J. Struct. Biol..

[9] Addadi L., Joester D., Nudelman F., Weiner S. (2006). Mollusk Shell Formation: A Source of New Concepts for Understanding Biomineralization Processes. Chem. Eur. J..

[10] Furuhashi T., Schwarzinger C., Miksik I., Smrz M., Beran A. (2009). Molluscan shell evolution with review of shell calcification hypothesis. Comp. Biochem. Physiol. B Biochem. Mol. Biol..

[11] Levi-Kalisman Y., Falini G., Addadi L., Weiner S. (2001). Structure of the nacreous organic matrix of a bivalve mollusk shell examined in the hydrated state using Cryo-TEM. J. Struct. Biol..

[12] Weiner S., Traub W. (1980). X-ray diffraction study of the insoluble organic matrix of mollusk shells. FEBS Lett..

[13] Weiss I.M., Renner C., Strigl M.G., Fritz M. (2002). A simple and reliable method for the determination and localization of chitin in abalone nacre. Chem. Mater..

[14] Agbaje O.B.A., Shir I.B., Zax D.B., Schmidt A., Jacob D.E. (2018). Biomacromolecules within bivalve shells: Is chitin abundant?. Acta Biomater..

[15] Giuffrea A.J., Hamm L.M., Hana N., De Yoreo J.J., Dove P.M. (2013). Polysaccharide chemistry regulates kinetics of calcite nucleation through competition of interfacial energies. Proc. Natl. Acad. Sci. USA.

[16] Arias J.L., Fernández M.S. (2008). Polysaccharides and proteoglycans in calcium carbonate-based biomineralization. Chem. Rev..

[17] Suzuki M., Saruwatari K., Kogure T., Yamamoto Y., Nishimura T., Kato T., Nagasawa H. (2009). An acidic matrix protein, Pif, is a key macromolecule for nacre formation. Science.

[18] Bahn S.Y., Jo B.H., Hwang B.H., Choi Y.S., Cha H.J. (2015). Role of Pif97 in Nacre Biomineralization: In Vitro Characterization of Recombinant Pif97 as a Framework Protein for the Association of Organic−Inorganic Layers in Nacre. Cryst. Growth Des..

[19] Montagnani C., Marie B., Marin F., Belliard C., Riquet F., Tayalé A., Zanella-Cléon I., Fleury E., Gueguen Y., Piquemal D. (2011). Pmarg-Pearlin is a Matrix Protein Involved in Nacre Framework Formation in the Pearl Oyster *Pinctada margaritifera*. ChemBioChem.

[20] Jin C., Zhao J., Pu J., Liu X., Li J. (2019). Hichin, a chitin binding protein is essential for the self-assembly of organic frameworks and calcium carbonate during shell formation. Int. J. Biol. Macromol..

[21] Arroyo-Loranca R.G., Hernandez-Saavedra N.Y., Hernandez-Adame L., Rivera-Perez C. (2020). Ps19, a novel chitin binding protein from *Pteria sterna* capable to mineralize aragonite plates in vitro. PLoS ONE.

[22] Treccani L., Mann K., Heinemann F., Fritz M. (2006). Perlwapin, an Abalone Nacre Protein with Three Four-Disulfide Core (Whey Acidic Protein) Domains, Inhibits the Growth of Calcium Carbonate Crystals. Biophys. J..

[23] Mann K., Siedler F., Treccani L., Heinemann F., Fritz M. (2007). Perlinhibin, a Cysteine-, Histidine-, and Arginine-Rich Miniprotein from Abalone (*Haliotis laevigata*) Nacre, Inhibits In Vitro Calcium Carbonate Crystallization. Biophys. J..

[24] Joubert C., Piquemal D., Marie B., Manchon L., Pierrat F., Zanella-Cléon I., Cochennec-Laureau N., Gueguen Y., Montagnani C. (2010). Transcriptome and proteome analysis of *Pinctada margaritifera* calcifying mantle and shell: Focus on biomineralization. BMC Genom..

[25] Kinoshita S., Wang N., Inoue H., Maeyama K., Okamoto K., Nagai K., Kondo H., Hirono I., Asakawa S., Watabe S. (2011). Deep Sequencing of ESTs from Nacreous and Prismatic Layer Producing Tissues and a Screen for Novel Shell Formation-Related Genes in the Pearl Oyster. PLoS ONE.

[26] Marie B., Joubert C., Tayaléa A., Zanella-Cléonc I., Belliarda C., Piquemald D., Cochennec-Laureau N., Marin F., Gueguen Y., Montagnani C. (2012). Different secretory repertoires control the biomineralization processes of prism and nacre deposition of the pearl oyster shell. Proc. Natl. Acad. Sci. USA.

[27] Takeuchi T., Kawashima T., Koyanagi R., Gyoja F., Tanaka M., Ikuta T., Shoguchi E., Fujiwara M., Shinzato C., Hisata K. (2012). Draft Genome of the Pearl Oyster *Pinctada fucata:* A Platform for Understanding Bivalve Biology. DNA Res..

[28] Marie B., Arivalagan J., Mathéron L., Bolbach G., Berland S., Marie S., Marin F. (2017). Deep conservation of bivalve nacre proteins highlighted by shell matrix proteomics of the Unionoida *Elliptio complanata* and *Villosa lienosa*. J. R. Soc. Interface.

[29] Mann K., Cerveau N., Gummich M., Fritz M., Mann M., Jackson D.J. (2018). In-depth proteomic analyses of *Haliotis laevigata* (greenlip abalone) nacre and prismatic organic shell matrix. Proteome Sci..

[30] Liao Z., Jiang Y.-T., Sun Q., Fan M.-H., Wang J.-X., Liang H.-Y. (2019). Microstructure and in-depth proteomic analysis of *Perna viridis* shell. PLoS ONE.

[31] Song X., Liu Z., Wang L., Song L. (2019). Recent Advances of Shell Matrix Proteins and Cellular Orchestration in Marine Molluscan Shell Biomineralization. Front. Mar. Sci..

